# Enhancing Self Care Among Oral Cancer Survivors Using a Digital Approach: The Empowered Survivor Trial

**DOI:** 10.1002/pon.70579

**Published:** 2026-08-26

**Authors:** Sharon L. Manne, Shawna V. Hudson, Janet H. Van Cleave, Marissa Grosso, Sara Frederick, Morgan Pesanelli, Neetu Singh, Justin Solleder, Denalee M. O'Malley, Elizabeth Handorf, Shou‐En Lu

**Affiliations:** ^1^ Rutgers Cancer Institute New Brunswick New Jersey USA; ^2^ Rutgers Robert Wood Johnson Medical School New Brunswick New Jersey USA; ^3^ UT Health Cizik School of Nursing Houston Texas USA; ^4^ School of Public Health Rutgers University New Brunswick New Jersey USA; ^5^ Merck Pharmaceuticals Rahway New Jersey USA

**Keywords:** cancer survivorship, digital interventions, oral cancer, self‐efficacy, self‐management

## Abstract

**Background:**

Oral and oropharyngeal cancer survivors experience debilitating physical and psychosocial challenges. Little knowledge exists on the efficacy of interventions to enhance self‐efficacy in managing these challenges, increase survivorship preparedness, and improve health‐related quality of life (HRQoL).

**Methods:**

Individuals (*n* = 643) diagnosed with oral or oropharyngeal cancer diagnosed within past 3 years were randomized to a digital intervention, Empowered Survivor (ES) or a Generic Online Intervention (GO). Primary (self‐efficacy, preparedness, HRQoL) and secondary outcomes (self‐care activities) were measured at Baseline, 2‐months, and 6‐months.

**Results:**

Participants assigned to ES reported greater self‐efficacy and increased self‐care activities of oral self‐exams, swallowing, and mobility exercises than those assigned to GO (self‐efficacy: 2 months, *p* = 0.003, 6‐months, *p* < 0.001; self‐care activities).

**Conclusions:**

The ES enhanced self‐efficacy and increased self‐care activities. Further examinations of survivorship preparedness and HRQoL in oral and oropharyngeal cancer are warranted.

**Trial Registration:**

Registered on clinicaltrials. gov as NCT04713449

## 1 Introduction

There is a growing population of oral and oropharyngeal cancer survivors in the US, particularly among survivors with HPV‐associated cancer [1], who have a much higher survival rate and longer disease‐free survival rate. The incidence of oral and oropharyngeal cancers is three times higher among men, making it the 8th leading cancer among men. There are known disparities in outcomes. Black patients have lower overall survival and disease‐free survival, and they are more likely to present with distant metastases and unresectable tumors [2]. Latinas are more likely to present with later‐stage disease [3].

The location of these cancers and its treatment can result in debilitating physical, functional, and psychosocial changes, which reduce the ability to swallow, taste, speak, eat, result in shoulder and neck discomfort, dental issues, and psychosocial concerns [4]. Once active treatment is complete, daily responsibility for self‐care of symptom management falls largely on the survivor. Because 20% of survivors develop a recurrence or metastatic disease within the first 2 years after treatment, recommended Follow‐up regimen includes ongoing surveillance [4, 5, 6]. Regular self‐examinations to locate suspicious areas in the mouth and neck are recommended [7], as lesions that are not evident on scans can be detected earlier and checked by a professional, reducing morbidity and mortality [8]. Engagement in exercises to improve or maintain the ability to swallow [9, 10] and maintain muscle strength are also recommended. Adherence to this self‐care regimen is less than optimal: 30%–50% do not adhere to Follow‐up appointments, and up to 80% do not adhere to oral care or swallowing recommendations [11].

Due to the self‐care burden, these survivors experience information and support needs, including the desire for more information regarding long‐term effects, recommended Follow‐up, how to recognize suspicious symptoms, and managing psychological concerns [12]. Many survivors report less self‐efficacy in their ability to manage their care regimen [13, 14]. In addition to low self‐efficacy, survivors report compromised HRQOL [15] and low levels of preparedness. For this population, lower HRQOL is typically characterized by difficulties with oral functions (i.e., swallowing, salivating, dental issues) and adverse changes in physical appearance [16]. An individual's perception they are prepared for the survivorship phase after treatment is associated with, with higher perceived quality of care, symptom management self‐efficacy, and HRQOL [17]. Given the salience of HRQOL concerns and self‐management demands, both constructs were targets of the Empowered Survivor intervention.

Despite their self‐care burden, low self‐efficacy, compromised HRQOL, and low preparedness, there are few evidence‐based self‐management interventions for this population. To date, the few published studies have focused on single symptom pilot studies [14, 18, 19]. To address this gap, we developed and evaluated the feasibility and preliminary efficacy of a digital self‐management intervention for oral and oropharyngeal cancer survivors' care, called Empowered Survivor [7]. Our work was guided by the theoretical construct of self‐efficacy, defined as the belief in one's ability to execute actions to deal with a situation successfully. Bolstering self‐efficacy in survivorship care is a primary outcome and a key construct targeted by Empowered Survivor.

The purpose of this study is to evaluate the efficacy of a digital intervention, Empowered Survivor (ES), against generic online materials (GO) [20] developed for cancer survivors. We had three aims. The first aim was to evaluate the impact of ES versus GO on the primary outcomes of self‐efficacy in managing care, preparedness for managing survivorship, and HRQOL. We hypothesized that the participants randomized to ES would report greater improvements in self‐efficacy for managing their care, preparedness for survivorship, and HRQOL. The second aim was to evaluate five putative mediators for the ES intervention effects, which correspond to the targets of the intervention and self‐management theory: planning, information needs, support needs, activation, and fear of recurrence. The third aim was to evaluate moderators of ES's impact. We hypothesized that social determinants (non‐White race, lower education) and access to informational resources (did not receive a survivorship care plan) would impact ES use with survivors with social and informational challenges likely to benefit more from ES than other groups.

## 2 Methods

Eligibility and recruitment are described in Manne and colleagues [7]. Briefly, this is a prospective, randomized controlled trial comparing ES versus GO with three online assessments at Baseline and 2 and 6 months post‐Baseline. The majority were recruited from four state registries (Cancer Registry of Greater California, Iowa, New Jersey, Utah), with a small percentage recruited from Rutgers Cancer Institute. The eligibility criteria were: (1) age > 18 years, (2) diagnosed with a first primary oral cavity or oropharyngeal cancer in the last 3 years, (3) currently cancer‐free, but could have experienced a recurrence, (4) had computer access, and (5) able to read English. The study was approved by the Rutgers University IRB (Protocol # Pro2020000768) and listed on Clinicaltrials.gov (NCT04713449).

### 2.1 Interventions

A detailed description of the interventions is provided in Manne and colleagues [7, 21] and a summary of all study measures can be found in Table 1. ES has five modules: (1) Introduction, (2) Swallowing and Strength, (3) Oral Care, (4) Long‐term Follow‐up, and (5) Calm and Connect. Swallowing and Strength (Module 2) contains an assessment of swallowing difficulty, demonstrations of swallowing and strengthening exercises, lymphedema management, and goal setting. Oral Care (Module 3) reviews dry mouth causes, contains a symptom assessment, strategies to keep one's mouth moist, maintaining nutrition, and goal setting. Long‐term Follow‐up (Module 4) includes a video demonstration of oral self‐exams, goal‐setting exercise, personalized Follow‐up care plan, and recommendations for smoking cessation and alcohol intake reduction. Calm and Connect (Module 5) covers how emotions affect self‐care, emotional management strategies, and information about HPV‐related oral cancers. ES has three maintenance features: a “favorites” function that saves intervention content, a “Message Board,” and a goal tracker, where goals can be viewed and modified.

**TABLE 1:** Descriptive Information regarding study measures.

Variable	Description	Time point		
		BL	2 months	6 months
Demographic variables				
Sex	Male or female	X		
Age	Age in years	X		
Ethnicity	Hispanic or non‐hispanic	X		
Race	White, black, american indian/Alaska, asian, native Hawaiian/Pacific islander	X		
Education	Less than 8 years ‐graduate degree	X		
Marital status	Married/live with partner, divorced, separated, single	X		
Employment	Employed, unemployed, homemaker, student, retired, disabled	X		
Insurance	Private (PPO, HMO), public (medicare, medicaid)	X		
Medical variables				
Time since diagnosis	Time since initial diagnosis in years	X		
Type of cancer	Oral, oropharyngeal	X		
Recurrence status	Has cancer recurred? (yes/no)	X		
Treatments	Chemotherapy, radiation, surgery	X		
Medical comorbidities	Charlson scale [22] 21 items	X		
Treatment summary	Provided summary of treatment at end of active treatment (yes/no)	X		
Primary outcome variables				
Self‐care self‐efficacy	Level of confidence in the ability to manage dental care, proper nutrition, swallowing and speaking, neck and shoulder muscle flexibility/strength, Follow‐up appointments, provider communication, oral self‐exam, emotions, smoking, alcohol use, overall confidence [14]; 23 items; alpha = 0.88–0.95	X	X	X
Survivorship preparedness	Degree to which information was sufficient, helpful, comprehensive. Satisfaction with the quantity and way information provided [23]; 10 items; alpha = 0.94–0.95	X	X	X
Head and neck cancer‐related quality of life	European organization for research and treatment of cancer‐quality of life questionnaire‐head and Neck‐43 [24]; 43 items; assesses cancer side effects and symptoms; alpha = 0.95–0.96.	X	X	X
Secondary outcome variables				
Oral self‐exam	Comprehensive examination of the inside and outside of the mouth and neck in the last month (yes/no)	X	X	X
Swallowing exercises	Exercises to improve swallowing in past month (yes/no)	X	X	X
Jaw and neck strengthening exercises	Exercises to improve or maintain muscle strength in jaw or neck (yes/no).	X	X	X
Mediator variables				
Information needs	Desire for more information about various topics including oral cancer symptoms, self‐exams, and Follow‐up tests (yes/no) [25]	X	X	X
Planning	Degree to which a detailed plan has been made for oral cancer self‐care tasks and how to manage interference in plans [26, 27]; 8 items; alpha = 0.93–0.94.	X	X	X
Activation	Attitudes about adopting active role in care, adapted to assess head and neck cancer care [28] 12 items; alpha = 90–0.93.	X	X	X
Support needs	Physical, psychological, and health care system needs [29]; 34 items; count of moderate and high support needs; alpha = 0.93–0.97.	X	X	X
Worry about recurrence	Worry about a recurrence of oral cancer [30]; 4 items; alpha = 0.92–0.93.	X	X	X
Intervention evaluation				
Treatment evaluation	*General* perceptions of intervention content (7 items); degree to which ES improved *confidence* in coping with aspects of survivorship (3 items), and perceptions of website *features* (e.g., ES: Goal setting; SB: action cards) (5 items); ES: number of log‐ins to website; SB: self‐reported			X

The generic online intervention consisted of two programs. The first program was Springboard Beyond Cancer, a general self‐management program developed by the ACS and NCI [31, 32]. The program covers symptoms, stress and mood, wellness, and how to get support. Each topic provides information on signs and symptoms, management tips, and links to resources. The NCI ended its contract with the ACS in 2020 and no longer hosted Springboard Beyond Cancer. The website was moved to archive status and dynamic features moved to YouTube. The second program was the ACS Survivorship: Before and After Treatment website [20], which includes information on coping with cancer, maintaining a healthy lifestyle, and managing cancer. Participants received the Springboard YouTube link in the welcome email. A week later, they received a second welcome email with links to ACS Survivorship website.

### 2.2 Sample

**FIGURE 1:**
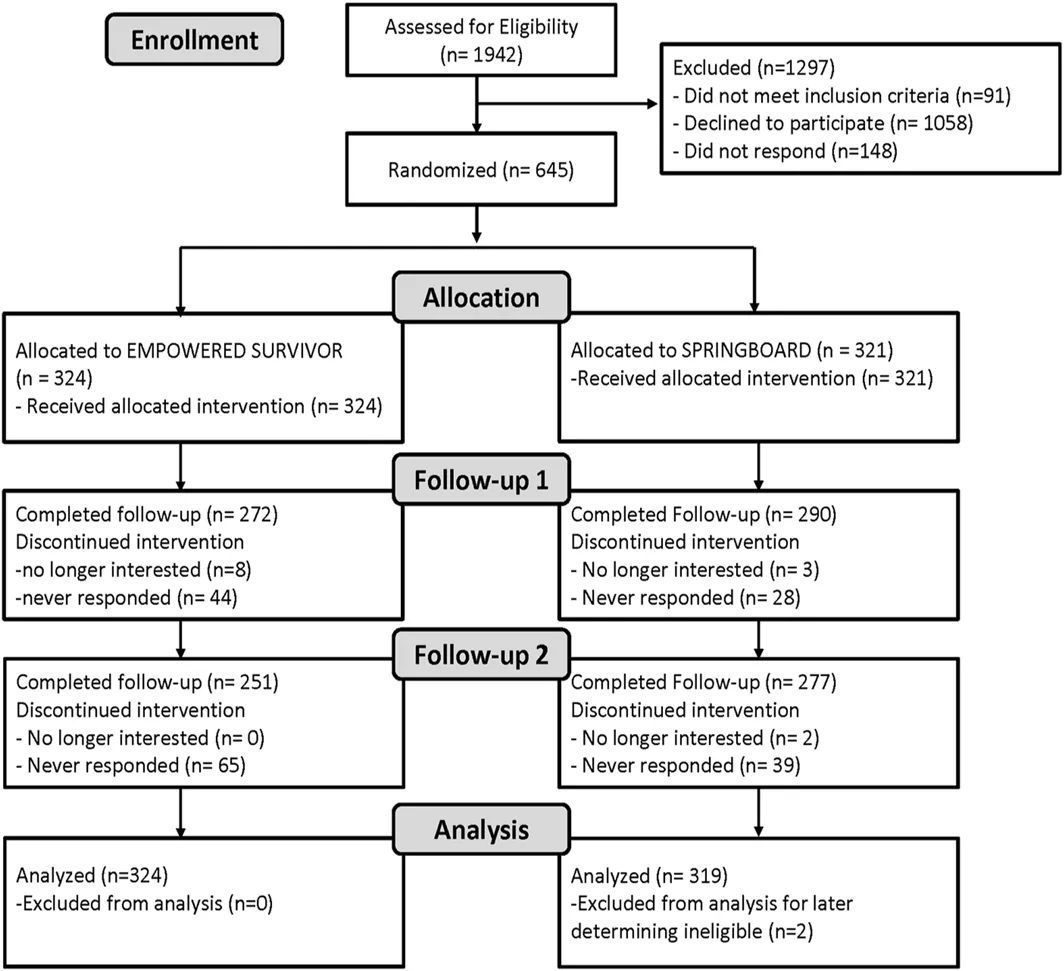
Study CONSORT.

The CONSORT is shown in Figure 1. Of 2006 patients approached, 91 were ineligible, 1122 refused, 148 could not be located or did not respond, and 645 accepted. The acceptance rate was 35.6 (645/1767). Two participants withdrew after consenting. Comparisons of the 643 participants with the 1122 refusers on available data indicated significant differences for age [*t* (df, 1421.8) = 4.75, *p* < 0.001, *M*_acceptor_ = 59.45, SD = 10.9, *M*_refuser_ = 62.1, SD = 11.8], race (41.0% White, 41.8% Black, 19.1% Asian, 78.6% multiple races, 4.5% unknown, *χ* ^2^ (df = 4) = 111.09, *p* < 0.001), time since diagnosis [*t* (df, 1393.7) = 26.24*, p* < 0.001, *M*_acceptor_ = 1.94 SD = 0.70, *M*_refuser_ = 2.89, SD = 0.72], and study site [13.7% California, 78.6% Iowa, 61.4% New Jersey, 26.7% Utah; *χ* ^2^ (df,3) = 423, *p* < 0.001]. Refusers were significantly older and more likely to identify as White race versus Asian race and have a longer time since their diagnosis. There were site differences (acceptance ranged from 78.6% to 13.7%). Return rates were higher for GO than ES. Return rates for Follow‐up 1 were 84% for ES and 89% for GO. For Follow‐up 2, return rates were 77% for ES and 86% for GO.

### 2.3 Statistical Analysis

Statistical analyses were performed on an intent‐to‐treat basis. We assessed intervention effects using linear mixed‐effects models with intervention arm, time, and the interaction of arm and time as fixed‐effect independent variables and a random intercept for subject. According to the pre‐defined analysis plan, we controlled for covariates (model fixed effects): site, age, gender, race, ethnicity, education, and stage of disease. Changes from the Baseline within the intervention arm and between the intervention arms were estimated and tested using linear contrasts. Sensitivity analyses for non‐ignorable missing data were conducted using the ISNI (Index of local Sensitivity to NonIgnorability). Moderation was assessed using 3‐way interaction terms between arm, time, and moderator variables. A significant Type 3 *F* test for the 3‐way interaction indicated evidence of moderation. For secondary outcomes, performance was categorized as binary. We used marginal logistic regression models for these outcomes, with the intervention arm, time, and the interaction between arm and time as the independent variables, and participant as subject unit, controlling for the same set of covariates as those in the linear mixed effects models. Statistical inference was using the generalized estimating equation approach with the robust sandwich variance estimates.

For mediation, we performed mixed model analyses following the principle in Hayes and Rockwood [33], to estimate the indirect effect and calculate the proportion mediated. We first assessed the relationship between each of the proposed mediators measured at 2 months and the treatment and performed the single mediator analysis to test the mediation effect on the 6‐month outcomes, followed by a parallel multiple mediator analysis including those shown a significant indirect effect. The mediation effect was tested using the 95% confidence interval obtained from the bootstrap method with 1000 samplings. Statistical significance was defined by *p* < 0.05. All statistical analyses were performed using SAS v.9.4 and R 4.4.3 [34].

## 3 Results

### 3.1 Sample Characteristics

The average age of participants was 62 years. Participants were primarily male, White race, non‐Hispanic ethnicity, relatively well‐educated, had an annual income of more than $140,000, and most carried medical insurance (Table 2). About half were diagnosed with oral cancer (48.5%), almost a third had been diagnosed with stage 3 or 4 cancer (30.6%), and a small fraction (5.3%) had a recurrence since the original diagnosis. There were no systematic differences between study arms.

**TABLE 2:** Demographic and medical characteristics of the sample.

Variable	Empowered survivor (*N* = 324)	Generic online (*N* = 319)	Full sample (*N* = 643)
	*N* (SD)	*N* (%)	*N* (%)
Age (years) [mean (SD)]	61.56 (11.06)	61.9 (10.92)	61.95 (10.92)
Sex			
Male	231 (71.3)	226 (70.8)	457 (71.1)
Female	93 (28.7)	93 (29.2)	186 (28.9)
Race			
White	278 (85.8)	274 (85.9)	552 (85.8)
Black	16 (4.9)	17 (5.3)	33 (5.1)
Asian	13 (4.0)	8 (2.5)	31 (3.3)
Native hawaiian/pacific islander	1 (0.3)	1 (0.3)	2 (0.3)
Multiple	9 (2.8)	13 (4.1)	22 (3.4)
Other/unknown	7 (2.1)	6 (1.8)	7 (1.1)
Ethnicity			
Non‐hispanic	307 (94.8)	297 (93.1)	604 (93.9)
Hispanic	15 (4.6)	19 (6.0)	34 (5.3)
Missing	2 (0.6)	3 (0.9)	5 (0.8)
Minority	103 (16.2)	50 (15.6)	53 (16.6)
Education			
Up to 12th grade	51 (15.8)	63 (19.7)	114 (17.8)
Some college/trade school	99 (30.6)	89 (27.9)	188 (29.2)
College degree or higher	173 (53.4)	166 (52.0)	339 (52.7)
Missing	1 (0.3)	2 (0.3)	2 (0.3)
Income			
< $30,000	34 (10.49)	36 (11.3)	70 (10.9)
$30,000–$74,999	89 (27.46)	76 (23.8)	165 (25.7)
$75,000–$139,999	95 (29.32)	96 (30.0)	191 (29.7)
$140,000 or more	103 (31.79)	106 (32.2)	209 (32.5)
Missing	3 (0.9)	5 (1.6)	8 (1.2)
Marital status			
Partnered	248 (76.6)	238 (74.6)	480 (74.6)
Not partnered	75 (23.1)	80 (25.1)	161(25)
Missing	1 (0.3)	1 (0.3)	2 (0.3)
Employment			
Employed	168 (51.9)	162 (50.8)	330 (51.3)
Not employed	155 (47.8)	134 (42.0)	311 (48.4)
Missing	1 (0.3)	1 (0.3)	2 (0.3)
Insurance			
Insured	318 (98.1)	317 (99.4)	635 (98.8)
Not insured	6 (1.9)	0 (0)	6 (0.9)
Missing	0 (0)	2 (0.6)	2 (0.3)
Cancer location			
Oral cavity	137 (42.3)	121 (37.9)	258 (40.1)
Oropharyngeal	187 (57.7)	197 (61.8)	384 (59.7)
Cancer stage			
0/1	147 (45.4)	150 (47.0)	297 (46.2)
2	73 (22.5)	57 (17.9)	130 (20.2)
3	50 (15.4)	35 (11.0)	85 (13.2)
4	49 (15.1)	51 (16.0)	112 (17.4)
Missing	5 (1.5)	14 (4.4)	19 (3.0)
Recurrence (yes)	18 (5.6)	18 (5.6)	266 (5.6)
Time since diagnosis (years)			
[Mean (SD)]	1.94 (0.70)	1.95 (0.70)	1.94 (0.70)
Treatments			
Surgery (yes)	211 (65.1)	196 (61.4)	429 (66.7)
Radiation (yes)	247 (76.2)	217 (68.0)	496 (77.1)
Chemotherapy (yes)	156 (48.1)	145 (45.5)	325 (50.5)
Comorbidities [mean (SD)]	0.73 (0.98)	0.75 (1.03)	0.74 (1.00)
Received treatment summary			
Yes	232 (71.6)	227 (71.2)	459 (71.4)
No	90 (27.8)	88 (27.6)	178 (27.7)
Unknown	2 (0.6)	4 (1.3)	6 (0.9)

### 3.2 Intervention and Moderator Effects

Although self‐efficacy significantly increased for both interventions over time, increase in self‐efficacy among participants in ES was significantly higher than in GO at both Follow‐ups (Follow‐up 1: difference = 0.13, 95% CI 0.05–0.22, *p* = 0.003; Follow‐up 2: difference = 0.15, 95% CI 0.06–0.24, *p* = 0.001) (Table 3). Sensitivity analyses for non‐ignorable missing data found these results to be robust, with sensitivity transformation c statistics > 1 (Follow‐up 1, *c* = 2.98, Follow‐up 2, *c* = 2.35). There were no significant ES effects on preparedness or HRQOL.

**TABLE 3:** Intervention effects on primary outcomes.

	Baseline	Follow‐up 1	Follow‐up 2	Changes from Baseline to Follow‐up 1		Changes from Baseline to Follow‐up 2	
	Mean (95% CI)	Mean (95% CI)	Mean (95% CI)	Mean difference (95% CI)	*p*‐value	Mean difference (95% CI)	*p*‐value
Self‐efficacy							
ES	3.65 (3.39, 3.92)	4.19 (3.92, 4.46)	4.22 (3.95, 4.49)	0.54 (0.48, 0.60)	< 0.001	0.56 (0.50, 0.62)	< 0.001
GO	3.70 (3.43, 3.96)	4.10 (3.84, 4.37)	4.11 (3.84, 4.38)	0.41 (0.35, 0.47)	< 0.001	0.41 (0.35, 0.47)	< 0.001
ES versus GO				0.13 (0.05, 0.22)	0.003	0.15 (0.06, 0.24)	0.001
Preparedness							
ES	3.08 (2.80, 3.36)	3.26 (2.98, 3.54)	3.36 (3.07, 3.64)	0.18 (0.10, 0.27)	< 0.001	0.28 (0.19, 0.37)	< 0.001
GO	3.05 (2.77, 3.33)	3.17 (2.89, 3.46)	3.26 (2.97, 3.54)	0.13 (0.04, 0.21)	0.003	0.21 (0.12, 0.29)	< 0.001
ES versus GO				0.06 (−0.06, 0.17)	0.353	0.07 (−0.05, 0.19)	0.251
HRQOL							
ES	25.69 (19.67, 31.71)	24.90 (18.87, 30.93)	24.12 (18.09, 30.16)	−0.80 (−1.67, 0.08)	0.074	−1.57 (−2.47, −0.67)	0.001
GO	26.29 (20.27, 32.31)	26.00 (19.97, 32.02)	25.24 (19.21, 31.26)	−0.30 (−1.15, 0.56)	0.500	−1.05 (−1.93, −0.18)	0.018
ES versus GO				−0.50 (−1.73, 0.73)	0.424	−0.51 (−1.77, 0.74)	0.422

*Note:* Results adjusted for study site, age, gender, race, ethnicity, education, and stage of disease.

Abbreviations: ES = Empowered Survivor; GO = Generic Online; HRQOL = Health‐related Quality of Life.

**FIGURE 2:**
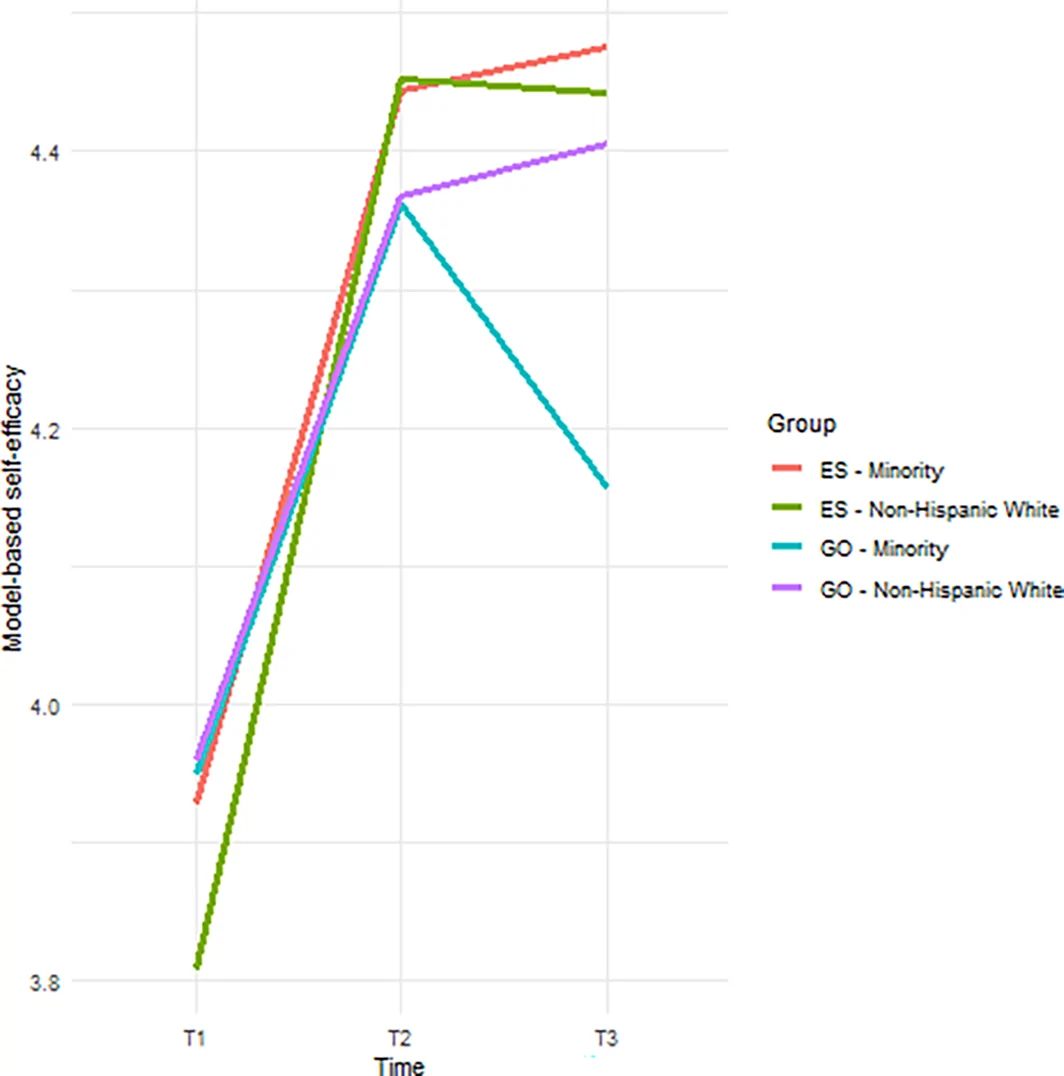
Race/ethnicity as a moderator of treatment effects.

In terms of moderation, the intervention effect was moderated by race (F(2,1057) = 3.51, *p* = 0.030). (see Figure 2). At Follow‐up 2, non‐White GO participants illustrated a significant decrease in self‐efficacy, whereas non‐White ES participants maintained a higher level of self‐efficacy. White patients had an increased self‐efficacy for both treatment arms. There was no significant moderation by survivorship care plan receipt, sex, or education.

### 3.3 Effects on Secondary Outcomes

Oral self‐exam performance increased in both ES and GO (Table 4). After controlling for covariates, the rate of oral self‐exam in ES increased from 56.0% at the Baseline to 79.4% at Follow‐up 1% and 80.1% at Follow‐up 2. In GO, the rate of self‐exam increased from 55.9% to 68.0% at Follow‐up 1% and 66.0% at Follow‐up 2. (OR = 3.03, 95% CI 2.24–4.10, *p* < 0.001 at Follow‐up 1 and OR = 3.16, 95% CI 2.30–4.34, *p* < 0.001 at Follow‐up 2, compared with Baseline). In GO, the rate of self‐exam increased from 55.9% to 68.0% at Follow‐up 1% and 66.0% at Follow‐up 2 (OR = 1.68, 95% CI: 1.31–2.16, *p* = 0.001, Follow‐up 1 and OR = 1.53, 95% CI 1.15–2.03, *p* = 0.001 at Follow‐up 2, compared with Baseline). The increase was significantly higher in ES than GO (Ratio of OR (ROR) of ES versus GO = 1.80, 95% CI 1.21–2.67 at Follow‐up 1; ROR = 2.06, 95% CI: 1.35–3.16 at Follow‐up 2).

**TABLE 4:** Intervention effects on secondary outcomes.

Outcome	Baseline Proportion (%) (95% CI)	Follow‐up 1 Proportion (%) (95% CI)	Follow‐up 2 proportion (%) (95% CI)	Follow‐up 1 versus Baseline Odds ratio or ROR^a^ (95% CI)	*p*‐value	Follow‐up 2 versus Baseline Odds ratio or ROR^a^ (95% CI)	*p*‐value
Oral self‐exam							
ES	56.0 (45.7, 65.8)	79.4 (71.1, 85.8)	80.1 (71.8, 86.4)	3.03 (2.24, 4.10)	< 0.001	3.16 (2.30, 4.34)	< 0.001
GO	55.9 (45.8, 65.5)	68.0 (58.3, 76.4)	66.0 (56.1, 74.6)	1.68 (1.31, 2.16)	< 0.001	1.53 (1.15, 2.03)	0.003
ES versus GO (ROR^a^)				1.80 (1.21, 2.67)	0.003	2.06 (1.35, 3.16)	0.001
Swallowing exercises							
ES	32.1 (24.3, 41.0)	56.1 (46.5, 65.2)	51.6 (41.8, 61.3)	2.70 (2.03, 3.61)	< 0.001	2.26 (1.70, 2.99)	< 0.001
GO	32.7 (25.0, 41.6)	41.1 (32.2, 50.7)	35.2 (26.8, 44.7)	1.43 (1.12, 1.84)	0.005	1.12 (0.84, 1.48)	0.439
ES versus GO (ROR^a^)				1.89 (1.29, 2.76)	0.001	2.02 (1.36, 3.00)	0.001
Muscle strengthening exercises							
ES	47.9 (38.0, 57.9)	71.9 (62.6, 79.7)	67.2 (57.1, 75.9)	2.79 (2.14, 3.65)	< 0.001	2.23 (1.67, 2.97)	< 0.001
GO	53.4 (43.4, 63.1)	57.1 (46.7, 66.9)	55.6 (45.4, 65.5)	1.16 (0.92, 1.47)	0.203	1.09 (0.85, 1.42)	0.493
ES versus GO (ROR^a^)				2.40 (1.69, 3.42)	< 0.001	2.04 (1.38, 2.99)	< 0.001

*Note:* Results adjusted for study site, age, gender, race, ethnicity, education, and stage of disease.

Abbreviations: ES = Empowered Survivor; GO = Generic Online.

^a^ ROR=Ratio of Odds Ratios.

Engagement in swallowing exercises increased in both groups, with significantly greater increases in ES than GO. In ES, engagement increased from 32% at Baseline to 56% at Follow‐up 1% and 52% at Follow‐up 2 (OR = 2.70, 95% CI 2.03–3.61, *p* < 0.001, Follow‐up 1, OR = 2.26, 95% CI 1.70–2.99, Follow‐up 2). In GO, engagement increased from 33% at Baseline to 41% at Follow‐up 1 (OR = 1.43, 95% CI: 1.12–1.84, *p* = 0.005) and to 35% at Follow‐up 2 (non‐significant OR = 1.12, 95% CI: 0.84–1.48, *p* = 0.439). The increase was significantly higher in ES than GO (ROR = 1.89, 95% CI 1.29–2.76, *p* = 0.001 at Follow‐up 1; ROR = 2.02, 95% CI: 1.36–3.00, *p* = 0.001 at Follow‐up 2).

Engagement in mobility exercises increased at a significantly greater rate in ES compared with GO. In ES, the rate increased from 47.9% at Baseline to 71.9% (OR = 2.79, 95% CI 2.14–3.65, *p* < 0.001) at Follow‐up 1% and to 67.2% at Follow‐up 2 (OR = 2.23, 95% CI 1.67–2.97, *p* < 0.001). For GO, the rate increased from 53.4% at Baseline to 57.1% at Follow‐up 1% and to 56% at Follow‐up 2 (OR = 1.16, 95% CI 0.92–1.47) and (OR = 1.09, 95% CI 0.85–1.42, respectively, both non‐significant *p* > 0.05). The increase was significantly higher in ES than GO (Follow‐up 1, ROR = 2.40, 95% CI 1.69–3.42, *p* < 0.001; Follow‐up 2, ROR = 2.04, 95% CI 1.38–2.99, *p* < 0.001).

### 3.4 Mediation Effects

**FIGURE 3:**
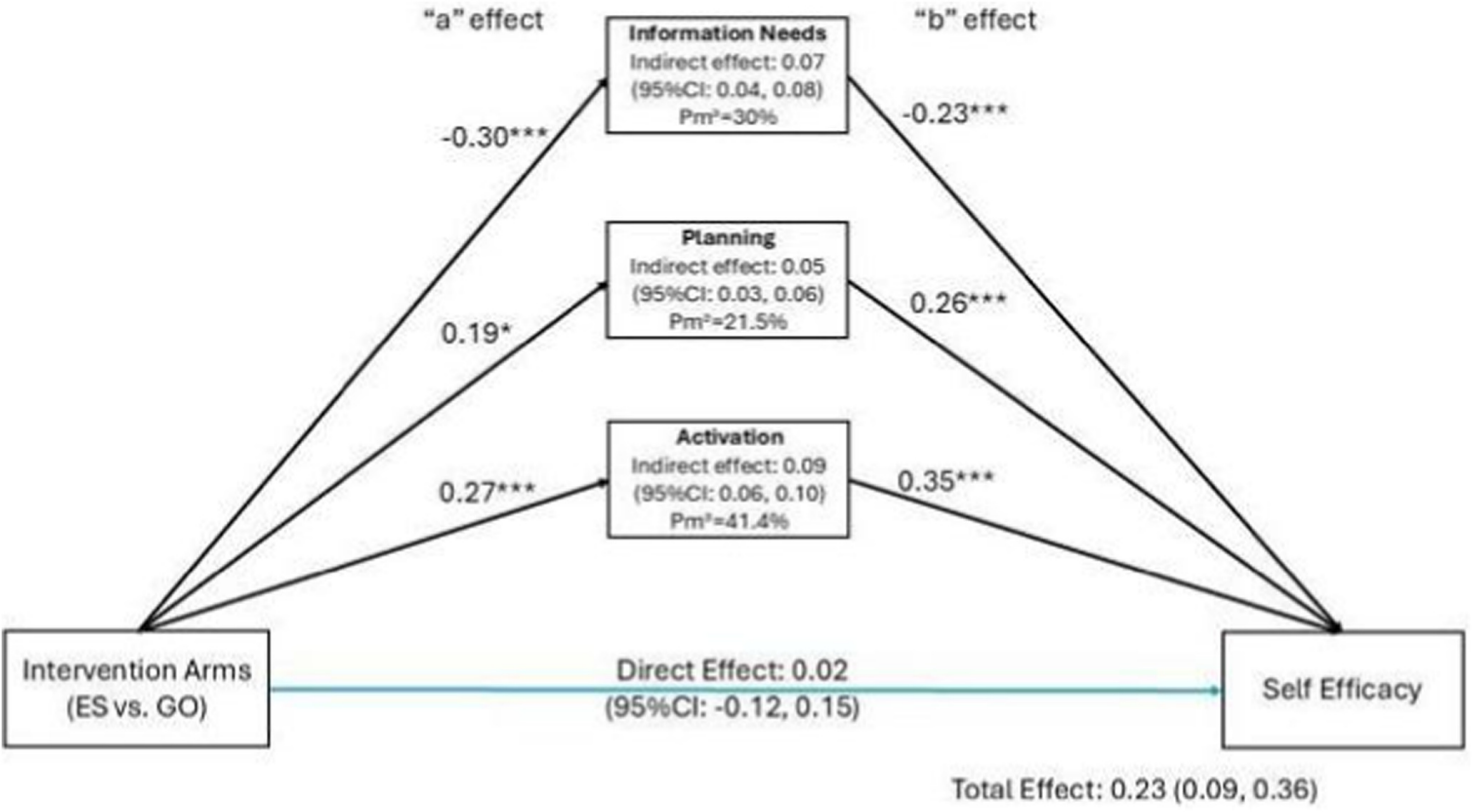
Mediation model. *p < 0.05; **p < 0.01; ***p < 0.001. ^a^PM = Proportion of Mediation.

Mediation was evaluated for self‐efficacy, as there were no significant effects on preparedness or HRQOL (Table 5). The single mediator analysis showed that the improvement of ES over GO in self‐efficacy at Follow‐up 2 was significantly mediated by information needs, planning, and activation at Follow‐up 1 [indirect effect = 0.10 (95% CI 0.05, 0.16), 0.09 (95% CI 0.01, 0.16) and 0.15 (95% CI 0.07, 0.23), respectively]. The parallel multiple mediator analysis showed that information needs, planning, and activation jointly mediated the improvement of ES over GO in self‐efficacy, with the indirect effect of 0.07 (95% CI 0.04, 0.08), 0.05 (95% CI 0.03, 0.06), 0.09 (95% CI 0.06, 0.10), respectively, with the portion mediated of 30.0%, 21.5%, 41.1%, respectively (Table 5 and Figure 3).

**TABLE 5:** Mediator effects on self‐efficacy.

	a effect		b effect		Indirect effect	Direct effect		Total effect	
	(95% CI)	*p*‐value	(95% CI)	*p*‐value	(95% CI)	(95% CI)	*p*‐value	(95% CI)	Proportion of mediation
Single mediator model									
Information needs	−0.30 (−0.45, −0.14)	< 0.001	−0.34 (−0.39, −0.29)	< 0.001	0.10 (0.05, 0.16)	0.10 (−0.04, 0.24)	0.170	0.20 (0.07, 0.34)	0.503
Support needs	−0.13 (−0.28, 0.01)	0.074	−0.35 (−0.40, −0.30)	< 0.001	0.05 (0.00, 0.10)	0.15 (0.01, 0.29)	0.034	0.20 (0.07, 0.34)	0.231
Planning	0.19 (0.03, 0.35)	0.019	0.47 (0.42, 0.51)	< 0.001	0.09 (0.01, 0.16)	0.12 (−0.02, 0.25)	0.089	0.21 (0.08, 0.34)	0.423
Activation	0.27 (0.13, 0.41)	< 0.001	0.55 (0.50, 0.59)	< 0.001	0.15 (0.07, 0.23)	0.08 (−0.06, 0.22)	0.285	0.23 (0.09, 0.36)	0.645
Fear of recurrence	−0.03 (−0.13, 0.08)	0.641	−0.32 (−0.37, −0.26)	0.000	0.01 (−0.03, 0.04)	0.19 (0.06, 0.33)	0.005	0.20 (0.07, 0.34)	0.04
Parallel mediation model									
Information needs	−0.30 (−0.45, −0.14)	< 0.001	−0.23 (−0.28, −0.19)	< 0.001	0.07 (0.03, 0.09)	0.02 (−0.12, 0.15)	0.779	0.23 (0.09, 0.36)	0.3
Planning	0.19 (0.03, 0.35)	0.019	0.26 (0.21, 0.30)	< 0.001	0.05 (0.01, 0.09)				0.215
Activation	0.27 (0.13, 0.41)	< 0.001	0.35 (0.30, 0.40)	< 0.001	0.09 (0.04, 0.13)				0.411

### 3.5 Intervention Evaluation and Utilization

Ratings were higher for ES than GO. The average general evaluation scale score was 5.6 for ES, 5.1 for Springboard, and 5.0 for ACS. The average confidence scale score was 5.2 for ES, 4.5 for Springboard, and 4.6 for ACS. The average features scale score was 5.2 for ES, 5.2 for Springboard, and 5.4 for ACS. It should be noted that the sample sizes were larger for the ES scales (*N* = 233–238) than Springboard (*N* = 188–191) and ACS scales (*N* = 76).

ES utilization indicated that 17.9% did not view any modules, 3.7% completed only Module 1, 4.3% completed only Modules 1 and 2, 5.2% completed only Modules 1–3, 4% completed only Modules 1%–4%, and 64.8% viewed all modules. For Springboard, self‐report indicated that 36.1% viewed most sections, all sections, or viewed it several or many times. For ACS, 34% viewed most sections, all sections, or viewed it several or many times.

## 4 Discussion

There have been few RCTs evaluating the efficacy of digital interventions targeting the broad array of self‐care needs among oral and oropharyngeal cancer survivors. In this large RCT recruiting recent survivors, we evaluated ES against a set of generic online materials. We evaluated short‐ and long‐term outcomes, as well as putative moderators and mediators. Survivors randomized to ES reported significantly higher increases in self‐efficacy and greater engagement in oral self‐exams, swallowing exercises, and head and neck mobility exercises as compared with survivors randomized to GO. These effects were maintained at the second Follow‐up.

To our knowledge, this study represents the only randomized clinical trial of an intervention for the oral and oropharyngeal cancer survivors targeting self‐management of a wide array of potential post‐treatment effects. The majority of published trials of digital health interventions have targeted selected symptoms such as trismus [35], swallowing [36, 37, 38], mucositis, pain, nutritional status [39], and social eating [40]. ES targets self‐management of many of these symptoms, but includes additional topics such as nutrition and dry mouth, psychological concerns, recommended Follow‐up, managing comorbid issues, tobacco and alcohol use, and goal setting. Our findings indicated a significant and sustained effect on self‐efficacy. Comparisons with other studies are hampered as self‐efficacy has not been incorporated as an outcome.

Both ES and GO improved preparedness, with no significant differences between the two interventions. It is difficult to make comparisons with the existing literature because preparedness has also not been included as an outcome. The one study evaluating preparedness included it as a moderator and reported inconsistent results [41]. There are at least two possible explanations for our findings. First, we did not use the same measure as the Stanton et al. study, which utilized two global assessments of how well the team prepared the patient for recovery. We did not directly assess general perceptions about preparation, which may have illustrated a differential impact in favor of ES. A second explanation is that both ES and GO impacted preparedness by providing basic information about expected symptoms and recommended Follow‐up, and that preparedness may be amenable to less intensive interventions.

It is unfortunate but not surprising that ES did not have a significant impact on HRQOL. Indeed, a recent review of digital interventions mixed results for their effects on HRQOL [42]. Because most interventions focus on specific symptoms and few interventions are digital, it is difficult to make comparisons. Wang and colleagues [43] conducted the only fully digital intervention study targeting this population. They developed a brief post‐surgical care intervention to provide information about oral cancer treatment, links to information about available support groups, as well as medical visit reminders. Results suggested a reduction in the EORTC QLQ‐HN35 subscales of sticky saliva, tube feeding, and weight gain, and physiological needs. One explanation is that the HRQOL measure assesses symptom burden. ES did not specifically target symptom relief, but rather more effective management of the symptom impact. Future work may benefit from using a measure of symptom interference, such as the M.D. Anderson Symptom Inventory scale.

The impact of ES on our secondary outcomes, particularly engagement in oral self‐exams, was impressive and sustained. To our knowledge, this is the only intervention trial that has included content illustrating how to perform an oral self‐exam. Effects on performance of swallowing exercises were also significant and sustained. This finding is consistent with the small swallowing exercise intervention literature illustrating an effect upon exercise adherence [44]. Our findings support three of the five proposed putative mediators: information needs, planning, and activation. This study is the first to identify mediators for a self‐management intervention for oral cancer, and our results bolster both the theoretical underpinnings of the intervention and the importance of promoting goal setting and self‐responsibility for self‐management in this population. It is unfortunate that concerns about recurrence did not mediate ES’ effects, given the prevalence of these fears and the inclusion of an entire module on self‐management of cancer‐related distress. It should be noted that there was no goal setting exercise in the Calm and Connect module, which may have improved its impact. Finally, the results suggesting that non‐White GO participants illustrated a significant decrease in self‐efficacy over the 6‐month Follow‐up, while non‐White ES participants maintained a higher level of self‐efficacy, are consistent with other m‐health and in‐person behavioral interventions [45, 46] for survivors with other cancers. Given the disparities in the incidence of adverse effects among Black survivors [2], a more tailored intervention may be needed for self‐efficacy for them to address disparities.

### 4.1 Strengths and Limitations

This study represents the largest randomized controlled trial to date examining the effects of a digital self‐management intervention for oral and oropharyngeal cancer patients and is the only trial focusing on recent survivors of this type of cancer. Other strengths are the inclusion of behavioral outcomes such as oral self‐exam and the diversity in income, education, and cancer stage. Finally, there was a relatively high utilization and positive evaluation of ES, which likely enhanced its effects on self‐efficacy and our behavioral outcomes.

There are some limitations. First, ES mitigated some of the declines in self‐efficacy seen over time among non‐White survivors randomized to GO. However, these results should be interpreted cautiously, because there were only 100 non‐White participants and the study was not powered for this interaction. Second, we recruited relatively few minority participants. There are well‐documented disparities in oral cancer survival among Black patients diagnosed with oral cancers [2, 3, 47]. Future studies would benefit from including minority survivors. Third, there were significant differences between participants and refusers that may have influenced the interpretation of results. Future work might benefit from more targeted recruitment efforts for older survivors and those diagnosed later in the survivorship trajectory. Fourth, there was lower engagement in GO as compared with the ES intervention and was not evaluated as positively as ES. GO's impact may have been low because its functionality was compromised. Finally, survey return rates were lower in ES (Follow‐up 1: 7.8‐point difference, Follow‐up: 9‐point difference). Although sensitivity analyses suggested that intervention effects were not sensitive to missing data, this differential return rate should be considered.

### 4.2 Clinical Implications

This fully digital intervention could be offered to oral and oropharyngeal cancer survivors, particularly if a longer‐term impact is demonstrated in a future clinical trial. Potential implementation options include delivery via electronic medical record to patients who have recently completed treatment or via oncologists, nurse navigators, or survivorship providers. Decades of psychosocial multicenter trials and registry‐based recruitment have attempted to solve the efficacy problem regarding recruitment to establish evidence based interventions for low volume cancers; however, the implementation challenges have yet to be sufficiently addressed [48, 49, 50]. A recent cluster randomized trial that evaluated team versus technology‐based supportive care interventions found lack of organizational prioritization, a culture that doesn't value psychosocial support, and funding constraints hindered the adoption of evidence‐based supportive care interventions [51]. Programs such as ES should leverage implementation science to determine the strategies for broad adoption and scalability. The knowledge about the barriers to translation could inform adaptations of ES based on patient needs, resource availability, and clinical infrastructure to optimize scalability and broad adoption. For example, the ES program could be targeted for patients who have not engaged in oral self‐exams and recommended exercises could be considered or could be delivered by a non‐profit partner with a more centralized mission of improving psychosocial care.

## 5 Conclusions and Future Directions

This trial illustrated that a digital intervention delivered to recent oral and oropharyngeal cancer survivors is effective in enhancing self‐efficacy in managing survivorship care and engagement in oral self‐exams, swallowing exercises, and strengthening exercises. ES effects on self‐efficacy were, in part, due to reductions in information needs and increases in activation and planning. In addition, further evaluation of ES’ impact on minority survivors may be important due to the disparities that this survivor population experiences.

## Author Contributions

Project conceptualization and methodology, was done by authors S.L.M., S.V.H., J.H.V.C., and D.M.O.M. Investigation and data curation was done by authors M.G., S.F., M.P., N.S., and J.S. Project administration and supervision were performed by authors S.L.M., and S.F. Formal analysis, validation, and visualization were performed by authors E.H, and S.‐E.L. Original draft was written by authors S.L.M., E.H., and S.‐E.L. All authors reviewed and edited the paper prior to submission.

## Funding

NCI R01 CA240344 to Sharon Manne.

## Conflicts of Interest

The authors declare no conflicts of interest.

## Supporting information


Supporting Information S1

## Data Availability

The data that support the findings of this study are available from the corresponding author upon reasonable request.
