# Supplementary material for: Enhancing Self Care Among Oral Cancer Survivors Using a Digital Approach: The Empowered Survivor Trial
Source: Psychooncology. 2026 Aug 26;35(9):e70579. doi: 10.1002/pon.70579 (PMC13519139; doi:10.1002/pon.70579)
Supplement: Supplementary file 1 [file PON-35-e70579-s001.docx]

**Intervention Evaluation**

|  | **Empowered Survivor** | **Springboard** | **American Cancer Society** |
| --- | --- | --- | --- |
| **General Evaluation** | **M (SD)** | **M (SD)** | **M (SD)** |
| Scale average | 5.82 (1.14) | 5.17 (1.28) | 5.09 (1.28) |
| Presented in accurate manner ^a^ | 6.06 (1.22) | 5.79 (1.18) | 5.83 (1.15) |
| Helped educate about the best way to navigate survivorship ^b^ | 5.94 (1.32) | 5.16 (1.61) | 5.03 (1.50) |
| Helpful in increasing understanding about the survivorship phase ^b^ | 5.91 (1.29) | 5.04 (1.67) | 5.00 (1.52) |
| Learned something new ^c^ | 5.55 (1.57) | 4.75 (1.67) | 4.64 (1.61) |
| Information was interesting ^c^ | 6.63 (1.40) | 5.14 (1.49) | 5.14 (1.39) |
| Information was valuable ^c^ | 5.56 (1.51) | 4.95 (1.65) | 5.01 (1.61) |
| Information was valid ^c^ | 5.91 (1.39) | 5.40 (1.51) | 5.50 (1.49) |
| **Confidence in coping with:** | **M (SD)** | **M (SD)** | **M (SD)** |
| Scale average ^d^ | 5.24 (1.43) | 4.51 (1.67) | 4.51(1.57) |
| Dry mouth symptoms ^d^ | 5.19 (1.50) | 4.42 (1.59) | 4.49 (1.47) |
| Issues faced with head and neck cancer ^d^ | 5.25 (1.51) | 4.49 (1.63) | 4.60 (1.49) |
| Long-term survivorship of head and neck cancer ^d^ | 5.29 (1.52) | 4.62 (1.71) | 4.71 (1.59) |
| **Features** ^a^ | 4.75 (1.50) | 4.70 (1.49) | 4.63 (1.49) |

**Note**. ^a^1 = *not at all,* 7 =*definitely; ^b^* 1*= not at helpful, 7 = definitely helpful; ^c^*1*= strongly disagree,* 7 = *strongly agree; ^d^*1*= not at all confident,* 7 *= very confident*

**Visualization of Race/Ethnicity as a Moderator of Treatment Effects**
